# Evacuation During Hurricane Sandy: Data from a Rapid Community Assessment

**DOI:** 10.1371/currents.dis.692664b92af52a3b506483b8550d6368

**Published:** 2016-01-29

**Authors:** Shakara Brown, Hilary Parton, Cynthia Driver, Christina Norman

**Affiliations:** Bureau of Communicable Disease, New York City Department of Health and Mental Hygiene, New York, NY, USA; Bureau of Epidemiology Services, New York City Department of Health and Mental Hygiene, New York, NY, USA; Bureau of Mental Health, NYC Department of Health and Mental Hygiene, New York, NY, USA

## Abstract

Introduction: In anticipation of Hurricane Sandy in 2012 New York City officials issued mandatory evacuation orders for evacuation Zone A. However, only a small proportion of residents complied. Failure to comply with evacuation warnings can result in severe consequences including injury and death. To better ascertain why individuals failed to heed pre­-emptive evacuation warnings for Hurricane Sandy we assessed factors that may have affected evacuation among residents in neighborhoods severely affected by the storm.

Methods: Data from a mental health needs assessment survey conducted among adult residents in South Brooklyn, the Rockaways, and Staten Island from December 13-­18, 2012 was assessed. Several disasters related questions were evaluated, and prevalence estimates of evacuation and evacuation timing by potential factors that may influence evacuation were estimated. Measures of association were assessed using chi-­square and t-­test.

Results: Our sample consisted of 420 residents of which, only 49% evacuated at any time for Sandy. Evacuation was higher among those who witnessed trauma to others related to the World Trade Center attacks (66% vs. 40%, p=0.024). Those who reported extensive household damage after Sandy, had a higher rate of evacuation than those with minimal damage (83% vs. 30%, p<0.001). Among those who evacuated, evacuation before the storm was lower among residents living on higher floors (56% vs. 22%, p=0.022).

Discussion: Given that warnings to evacuate were issued before Sandy made landfall, evacuation among residents in South Brooklyn, the Rockaways and Staten Island, while higher than the overall Zone A evacuation rate, was less than optimal. Continued research on evacuation behaviors is needed, particularly on how timing affects evacuation. A better understanding may help to reduce barriers, and improve evacuation compliance.

## Introduction

In 2012 Hurricane Sandy inundated parts of New York City (NYC) and left millions of residents without power, displaced many from their homes, and resulted in at least 43 deaths.^1^^,^ 2 ^,^ 3 ^,^ 4 Before the storm, city officials issued mandatory evacuation orders to thousands of residents, but few complied.^1^^,^ 2 ^,^ 5 Evacuation is important for reducing injury and mortality during disasters. However, people’s decisions to evacuate are likely influenced by multiple factors.^5^^,^ 6 ^,^ 7 ^,^ 8 ^,^ 9 ^,^ 10 ^,^ 11 ^,^ 12 ^,^ 13 ^,^ 14 ^,^ 15 ^,^ 16 In an effort to understand why residents did not comply with evacuation orders we assessed factors that may have affected evacuation among NYC residents severely affected by Sandy.

## Methods

The NYC Department of Health and Mental Hygiene (DOHMH) conducted a mental health needs assessment among adult residents in South Brooklyn, the Rockaways, and Staten Island approximately six weeks after Sandy. Sixty-two U.S. census block groups (i.e. clusters) were randomly chosen using probability proportional to size sampling (PPS) with replacement, from all block groups in selected inundation areas including, neighborhood tabulation areas (NTAs) of Coney Island, West Brighton, Gravesend, Brighton Beach, Sheepshead Bay, the Rockaways, and Staten Island. Seven tax lots were also randomly chosen from each cluster using PPS, and one interview from each primary address at a tax lot was completed. In-person surveys were administered by DOHMH staff from December 13 -16, and December 18, 2012. The NYC DOHMH Institutional Review Board determined the original study to be public health surveillance, and all secondary analyses, including this study, were deemed as not constituting research involving human subjects. Informed consent was obtained from all participants prior to survey administration. At the end of data collection, there were 420 completed surveys (45% of all eligible households). Data from completed surveys was used in this analysis.

Evacuation was self-reported by respondents, and responses were categorized to determine evacuation before, during, or after the storm. Evacuation was collapsed into a dichotomized (yes/no) variable, and evacuation timing was assessed comparing those who evacuated either during or after the storm to those who evacuated before. Demographic factors included gender, age group, employment status, race/ethnicity, home ownership, and poverty level. Additional measures assessed included household damage (none or minimal, damaged but livable, or damaged unlivable/destroyed) number of people in the household (1-2, 3-4, ≥5), child in the home under 18 years (yes/no), child age group (≤5, 6-11, and 12-17 years), number of children under 18 years in the home (none, 1 or 2, ≥3), and apartment level based on floor of residence (1st - 2nd, 3rd - 5th, and 6th or higher). Prior trauma exposure was defined based on responses to the following questions: “Not including things that happened during the storm, did something terrible ever happen to you so that you thought you might get hurt very badly or killed?” and if Yes, “Was this related to the events of September 11, 2001?” Responses to trauma questions were combined to create separate dichotomous variables for 9/11 if 9/11 related, and any trauma to self/others.

Significance (P<0.05) testing of bivariate associations was assessed using a chi-square test for selected factors and evacuation status. For the outcome, evacuation before Sandy, only significant variables in bivariate analyses were further assessed using t-tests for comparisons of proportions. Analyses were weighted at the household, individual, and child levels to account for survey participation by cluster, probability of selection from varying household sizes, and non-response by age and sex. Analyses were performed using SAS version 9.2 and SUDAAN version 11.0.1.

## Results

Respondents were predominantly female (54%), middle aged (25-64: 70%), employed (52%), and white non-Hispanic (60%) Forty nine percent of residents evacuated at any time for Sandy. Of these 24% evacuated before, 11% evacuated during, and 14% evacuated after the storm (Table 1).

**Figure:**
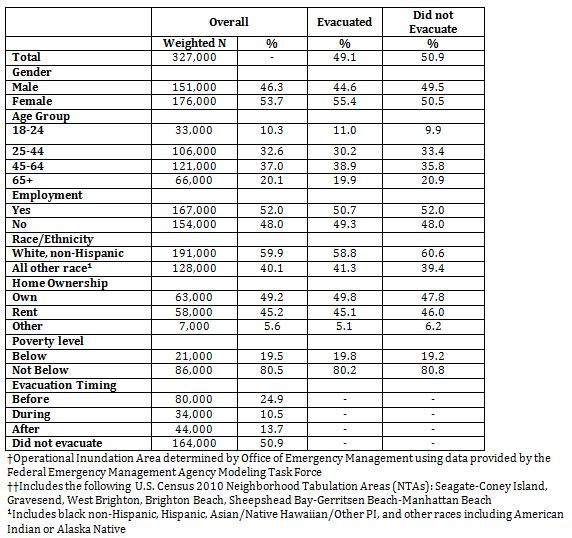
**Table 1: Demographic characteristics among adults living in households in the inundation zone†, South Brooklyn††/Rockaways, and Staten Island by evacuation status**

Table 2 shows selected factors that may have influenced evacuation behaviors and evacuation timing. No differences in rates of evacuation were observed by demographic characteristics. Compared to those with little to no damage, those who reported extensive household damage following Sandy had a higher rate of evacuation (83% vs 30%, p<0.001). And those witnessing trauma to others related to the World Trade Center attacks (9/11) were more likely to evacuate for Sandy than those who did not witness trauma (66% vs. 40%, p=0.024). Apartment level was the only variable significantly associated with evacuation timing. Among evacuees, individuals living on the first or second floor were more likely to evacuate before the storm compared to those on floor six or higher (56% vs. 22%, p=0.022).

**Figure:**
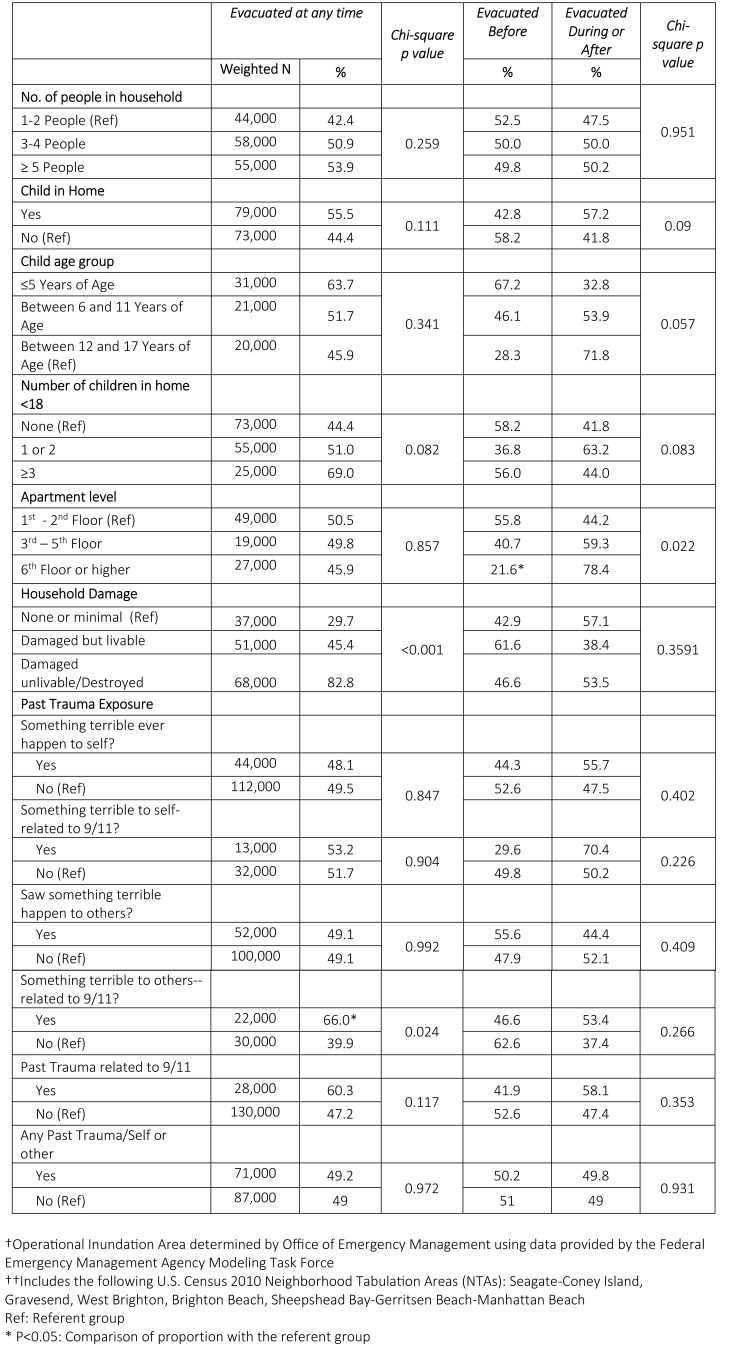
**Table 2: Prevalence of evacuation among adults living in households in the inundation zone†, South Brooklyn††/Rockaways, and Staten Island**

## Discussion

Less than half of survey respondents evacuated for Sandy. We consider this to be low considering that residents in areas more vulnerable to Sandy were instructed to evacuate. Possible explanations for this low evacuation rate may be due to several factors. A number of disaster studies have assessed how individuals respond to disaster warnings, and in most cases the timing, personalization, and clarity of the message, and risk perception affected evacuation.^7^^,^ 9 ^,^ 10 ^,^ 15 ^,^ 17 It is possible that the degree of evacuation warning compliance in this study was a result of these underlying dimensions, which were beyond the scope of this study.

The finding that individuals with extensive household damage were more likely to have evacuated than those with little to no damage is not entirely surprising. The evacuation rate previously reported for residents in evacuation zone A was only 31%.^5^ When taken into consideration with the high rate of evacuation among those with household damage, it is possible that respondents chose to evacuate because their homes became unlivable. Residents may not have fully grasped the necessity of evacuating prior to Sandy’s approach.

Research suggests that recollections of past trauma may increase vulnerability to adverse outcomes during disasters.^18^ Thus, we assumed that those who had prior trauma would be more willing to evacuate for Sandy. However, in our study only witnessing a traumatic 9/11 event to others was associated with greater likelihood of evacuation. Various degrees of trauma or trauma type might have differing impacts on evacuation behaviors during disasters. Future studies are needed to further assess this relationship and to ascertain to what extent witnessing a traumatic event influences evacuation compliance.

Notably, in this study, we found that individuals living on the first or second floor were more likely to evacuate before the storm compared to those on floor six or higher. In high-rise buildings, unlike those on higher floors, residents on lower floors are more susceptible to flooding during storms. The observed difference in evacuation timing between these two groups could be attributed to the perception of immediate flood risk. Residents on lower floors might be more partial to timely evacuation because they have a higher perceived risk of flooding.

These results are subject to several limitations. Lack of statistical significance and reliability of some estimates may be due to small cell sizes. Additionally, the survey did not collect information on reasons for evacuation, preventing us from further assessing the relationship between several other factors and evacuation. Certain items on the questionnaire were not specific enough to elicit meaningful responses that could be incorporated in our analysis. Future surveys could enhance our understanding of people’s behaviors leading up to and during evacuation situations by including more nuanced questions on evacuation reasons.

Despite these limitations, we were able to evaluate evacuation behaviors among a population residing in areas that were severely affected by Hurricane Sandy. It is clear from our findings that, even with pre-emptive warning, evacuation compliance was low among individuals residing in areas severely affected by Sandy. Evacuation behaviors are complex and thus, require continued exploration, especially those pertaining to evacuation timing. Our findings demonstrate how certain factors can affect evacuation at differing times. Recurrent research studies are needed to further assess timing of evacuation, as well as other ongoing and emerging barriers to compliance. And more comprehensive disaster messaging to residents informing them of the dangers associated with underestimating storm impact are needed, and might prove beneficial to those least likely to evacuate.

## Competing Interests

The authors have declared that no competing interests exist.
